# Use of burosumab in McCune Albright syndrome: case report and review of literature in mosaic disorders with FGF23 overproduction

**DOI:** 10.3389/fendo.2025.1577734

**Published:** 2025-05-26

**Authors:** Alessandro Barbato, Renato Vaiasuso, Eugenio Trinati, Giulia Del Medico, Nicolò Chiti, Giampiero Igli Baroncelli, Stefano Stagi

**Affiliations:** ^1^ Department of Health Sciences, University of Florence, Florence, Italy; ^2^ Auxo-endocrinology Unit, Meyer Children’s Hospital IRCCS, Florence, Italy; ^3^ Pediatric and Adolescent Endocrinology, Division of Pediatrics, Azienda Ospedaliero-Universitaria Pisana, Pisa, Italy

**Keywords:** McCune Albright syndrome, fibrous dysplasia, cutaneous-skeletal hypophosphatemia syndrome, burosumab, phosphate metabolism, FGF23

## Abstract

Increased fibroblast growth factor 23 (FGF23) related mosaic syndromes include a spectrum of disorders sharing postzygotic mutations, skin involvement and dysplastic bone lesions. This group encompasses both McCune Albright syndrome (MAS) and cutaneous-skeletal hypophosphatemia syndrome (CSHS). The altered production of FGF23 contributes to progression of the typical bone lesions of these disorders through a constant disruption of phosphate wasting and bone metabolism. In pediatric age, the current therapeutic strategies for fibrous dysplasia (FD) are able to control pain and reduce the entity of disability but not to improve disease course. FGF23 production is increased in MAS and negatively influences phosphate levels and bone metabolism. The availability of burosumab, an anti FGF23 antibody, introduced a potential new therapeutic tool for children with FD. A narrative review concerning the use of burosumab in MAS and CSHS was performed and the midterm outcome of treatment with burosumab in a 11-year-old patient with MAS and severe FD was described. The patient referred to our Center for periodic follow-up and treatment of severe FD. He was diagnosed with FD at the age of 1 year and 8 months and underwent four pathological fractures and two surgical interventions for correction of bone deformities. At the age of 5 years and 6 months, intravenous neridronate was started every 3 months with a partial improvement of bone pain and bone deformities. At the age of 8 years 9 months, subcutaneous periodic infusions of burosumab were started. Before treatment, laboratory assessment showed increased levels of FGF23 and alkaline phosphatase (ALP), and reduced phosphate with normal parathyroid hormone (PTH) levels. After 1 year of treatment with burosumab, a normalization of phosphate, ALP reduction, and normal to slightly increased PTH were observed. Nonetheless, a partial progression of FD was documented on periodic X-rays. Burosumab showed beneficial effects on bone tissue metabolisms in our patient without significant adverse effects but did not change FD course.

## Introduction

Fibroblast growth factor 23 (FGF23) related mosaic syndromes include a spectrum of disorders sharing postzygotic mutations, overproduction of FGF23, skin involvement and dysplastic bone lesions. Patients with McCune Albright syndrome (MAS; OMIM #174800) and cutaneous-skeletal hypophosphatemia syndrome (CSHS) are associated with an increased production of FGF23 that contributes to progression of the bone lesions through an impaired phosphate and bone metabolism (1).

MAS is a sporadic genetic condition associated with bone fibrous dysplasia (FD), café au lait skin macules and multiple endocrine over-function, including peripheral precocious puberty (PPP), hyperthyroidism, growth hormone excess and Cushing syndrome. It is caused by postzygotic mosaic activating mutations of *GNAS* gene located on chromosome 20q13.3, leading to increased activity of Gsα protein (2, 3).

FD is one of the key features of MAS and may cause bone deformities with pathologic fractures. FD is classified as monostotic if only one bone is affected, or polyostotic if multiple bones are involved (2–4). It may occur in many skeletal sites but mostly in proximal femur and basal skull (4, 5). The typical bone deformity associated with femoral involvement is the “sheperd’s crook”, a severe form of coxa vara with characteristic enlargement of bone tissue close to the femoral neck and sclerotic reactive bone around the lesion in the X-ray (rind sign) (4, 6). When FD involves the skull two main factors should be considered: the position of the lesion and the rate of progression of deformity, potentially leading to esthetic damage alone or to compression of structures of basicranium or hearing loss (7, 8).

The pathophysiologic milestones of FD are the activating mutations in *GNAS*, as they essentially impair the differentiation of stromal cells in bone marrow, leading to development of fibrous areas intertwined with disrupted bone tissue and without bone marrow (5, 9). Trabecular structure of the bone tissue in the affected areas is typically thinner and more fragile. Contributing factors to the reduced resistance of FD to mechanic stress are the increase of osteoclast activity and the osteomalacic rearrangements of bone matrix (5).

A persistent activation of the pathway of Gsα leads to increased production of FGF23, a main phosphatonin which regulates renal excretion of phosphate (10, 11). Overproduction of FGF23 is not exclusive of patients with MAS and may be observed in other conditions with FD (1). FGF23 overproduction may negatively influence bone metabolism in FD by inducing phosphate wasting and reducing 1,25-dihydroxyvitamin D (1,25(OH)_2_D) (10, 12). Although the increase of FGF23 is a common feature in patients with MAS, only the patients with extensive bone involvement are prone to develop hypophosphatemia (11). This finding may be explained by an imbalance between the production of FGF23 and its cleavage which is increased in patients with MAS leading to an increase of the inactive C-terminal FGF23 form (13). This process is considered one of the key determinants to the osteomalacic changes in FD bone, as it was observed a greater disease burden in patients with polyostotic FD and increased urinary phosphate excretion (5, 14).

Current treatment for patients with FD is focused on pain control and reduction of burden of disability but a specific therapy to modify the course of disease is still missing (15). In adult patients with FD, the administration of anti-RANK-L monoclonal antibody denosumab was proposed as potential treatment but some concerns about safety limit its use in pediatric age, especially for the risk of rebound hypercalcemia (16, 17). Burosumab is a monoclonal human monoclonal antibody inhibiting FGF23 approved for treatment of X-linked hypophosphatemia (18). Its pharmacodynamics make burosumab a potential treatment for patients with FD in whom the increased circulating FGF23 levels act as a precipitating factor on dysplastic bone areas.

Cutaneous-skeletal hypophosphatemia syndrome (CSHS) is a rare bone disorder featuring skeletal and skin manifestations caused by mosaic post zygotic activating variants of genes belonging to the RAS family (1). Similarly to MAS, CSHS is characterized by the overproduction of FGF23, secreted by osteocytes in affected dysplastic bones, resulting in hypophosphatemia, bone pain, rickets, long bone deformity, impaired growth and mobility (1). CSHS shows peculiar histologic features as dysplastic bone lesions do not replace bone marrow precursor cells and produces only focal areas of fibrous stroma, which alters the structure of trabecular and cortical bone (19, 20). Nonetheless, both FD and CSHS bone lesions share osteomalacic features, such as a reduction of the mineralized bone matrix (1).

## Methods

A review of the literature on burosumab treatment in FGF23 mosaic disorders was performed. Research articles were identified using the keywords: “fibrous dysplasia”, “McCune Albright syndrome”, “cutaneous-skeletal hypophosphatemia syndrome”, “epidermal nevus syndrome”, and “burosumab” on Pubmed for the period of time between 2000 and 2024.

We retrospectively also report the case of a 11 years old boy with MAS treated with burosumab for a non-continuous period of 18 months. The use of burosumab as compassionate treatment and the choice of treatment regimen were approved by our local Ethical Committee. Our study was conducted in respect of the declaration of Helsinki II. Written consent was obtained by the parents of the child.

## Case presentation

**Figure 1:**
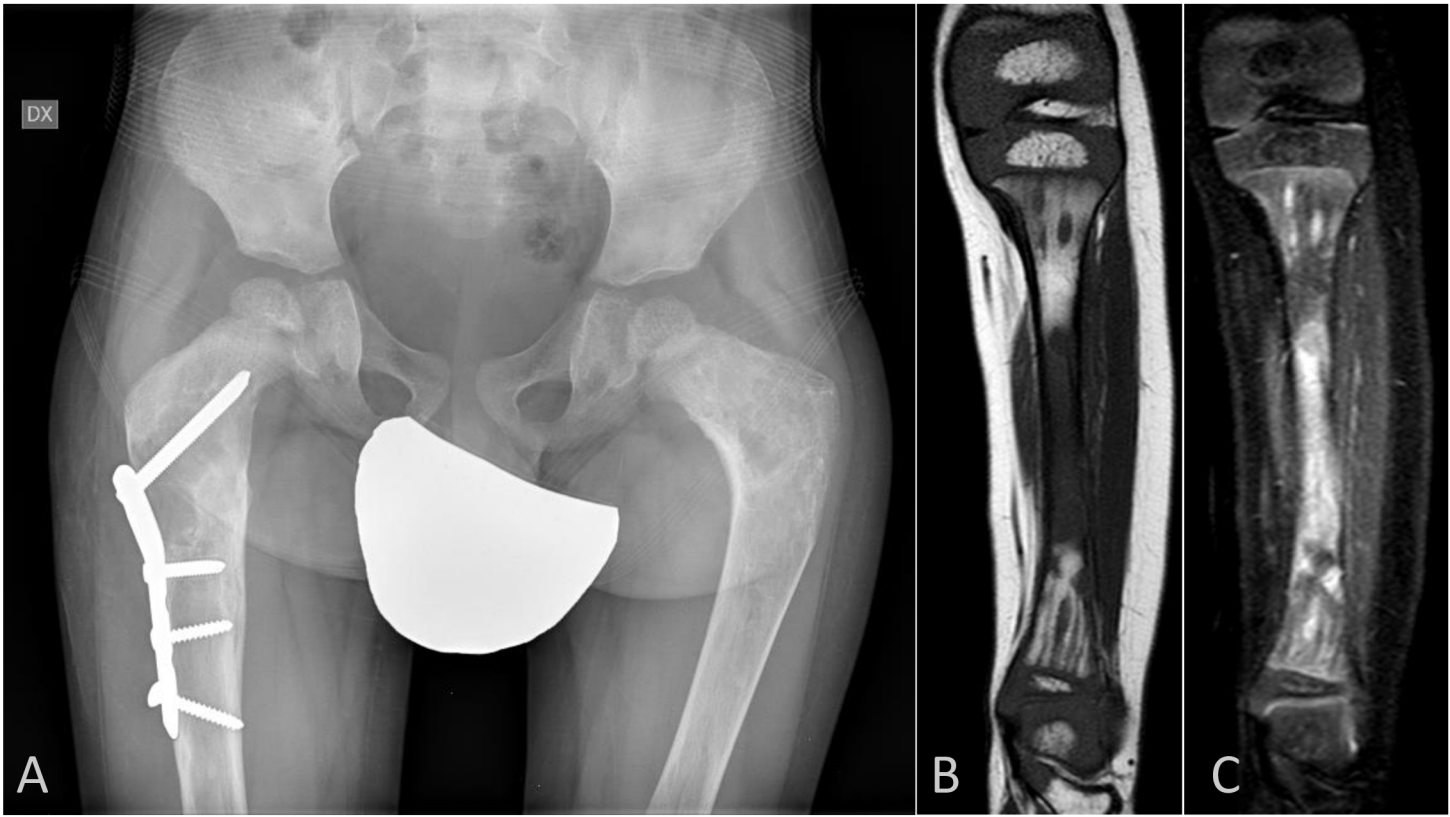
**(A)** sacrum X-ray performed at the age of 40 months; typical femoral neck enlargement (“sheperd’s crook”) associated with FD can be observed on both sides. **(B, C)** tibial MRI performed at the age of 33 months; in the image **(B)** signal hyperintensity associated with FD can be observed in the T1 sequence, especially in tibial diaphysis; in the image **(C)** the same section shows discontinuous signal hyperintensity in the short tau inversion recovery (STIR) sequence.

A 11-years-old boy was in follow-up in our Center for MAS complicated by severe FD. He was diagnosed with FD at the age of 1 year and 8 months meeting 2 diagnostic criteria for diagnosis of MAS (FD and characteristic café-au-lait skin pigmentation) (20). He developed a pathological fracture of distal left tibia at the age of 19 months suggestive of FD. A whole-body magnetic resonance imaging (MRI) at the age of 2 showed polyostotic FD involving both humerus, right radius, femurs, fibulae and tibiae, iliac crests, and right ischium. Genetic sequencing of the *GNAS* gene, performed at 1 year 8 months, showed mutation c.601C>T(p.Arg201Cys) in 5.39% of analyzed cells from a sample of bone from FD area. **Figure 1** shows the typical features of FD on the patient’s pelvis X-ray at the age of 40 months and MRI of the left leg performed at the age of 45 months. Up to the age of 11 years, four pathological fractures and two surgical interventions for correction of bone deformity were performed.

At the age of 2 years and 5 months, during the first endocrinological assessment, his height, weight, and BMI were -2.1 SD, -3.1 SD, and -2.8 SD, respectively, according to WHO growth curves for males. Laboratory assessment showed increased ALP (660 IU/L, age specific reference range 105.0- 280.0IU/L for males 1–10 years old) (21) and ALP bone isoenzyme (BALP, 283 μg/L, age specific reference range –24.5-169.5 μg/L for males 1–18 years old) (22); total serum calcium was 10.4 mg/dL (reference range 9.3 - 10.6 mg/dL), ionized calcium 4.5 mg/dl (reference range 4.3 - 5.3 mg/dL), serum phosphate 4.4 mg/dL (age specific reference range 3.8-6.5 mg/dL for children 1–3 years old) (23), PTH 25.3 pg/mL (reference range 12–60 pg/mL), 25-hydroxyvitamin D (25OHD) 49 ng/mL (reference range 20.0 – 100.0 ng/mL). Treatment with calcium carbonate (250 mg daily) and cholecalciferol (1000 IU daily) was started.

At the age of 4, testicular microcalcifications were found by ultrasound. Moreover, subclinical hyperthyroidism was diagnosed with TSH 0.02 mcUI/mL (reference range 0.40 - 4.00 mcUI/mL), FT_4_ 1.24 ng/dL (reference range 0.80 - 1.90 ng/dL), and FT_3_ 6.24 pg/L (reference range 2.91 - 4.70 pg/L). Because of the presence of bone pain and bone deformity, intravenous neridronate (2 mg/kg/every 3 months) was started at 5 years 6 months, with partial control of bone pain but progression of bone deformities. Before the start of neridronate, patient showed increased ALP values (up to 1228 IU/L) and increased FGF23 levels (144 pg/mL; reference range 23.2 - 95.4 pg/mL by DiaSorin, Saluggia, Italy), reduced serum phosphate levels (nadir 3.6 mg/dL) and tubular maximum reabsorption of phosphate to glomerular filtration rate ratio (TmPO_4_/GFR, 3.3 mg/dL; reference range 3.4 - 5.8 mg/dL) (24). Neridronate was chosen over other bisphosphonates considering the effects of intravenous bisphosphonates on pain control in FD (5) and its wide use in pediatric cases of osteoporosis and osteogenesis imperfecta in Italy (25).

Because of the clinical worsening of FD associated with worsening of biochemical markers of bone metabolism, neridronate treatment was suspended and, after three months (8 years 9 months of chronological age) subcutaneous burosumab treatment (0.4 mg/kg every two weeks) was started. At the start of burosumab treatment, laboratory assessments showed hypophosphatemia (3.1 mg/dL, age specific reference range 3.7 – 5.6 mg/dL for children 4–11 years old) (23), increased FGF23 level (251 pg/mL), ALP (2118 IU/L) and BALP levels (942 μg/L), normal 25OHD (58 ng/mL) and PTH levels (38 pg/mL), and reduced TmPO_4_/GFR values (2.8 mg/dL). The dosing regimen of burosumab was increased to 0.8 mg/kg every two weeks after one month of treatment.

At the age of 9, the patient developed signs of pubertal progression (testicular enlargement from 4 mL to 6 mL in 6 months); baseline serum testosterone levels were 10.6 ng/mL, LH was 0.9 mIU/mL and FSH was 2.4 mIU/mL. LHRH test (Lutrelef, 0.8 mg/mL; Ferring) showed a LH peak of 14.7 mIU/mL and an FSH peak of 5.9 mIU/mL. Diagnosis of central precocious puberty (CPP) was made and treatment with intramuscular triptorelin 3.75 mg every 28 days was started.

During the treatment with burosumab, laboratory tests showed a sustained improvement of phosphate metabolism after 6 months: ALP 1507 IU/L, BALP 623 μg/L, serum phosphate 4 mg/dL. Pain of the lower limbs, assessed by the Numeric Pain Rating Scale (NPRS), was well controlled during treatment with a value of 0, compared with the values assessed before burosumab that ranged from 2 to 6. The severity of femoral deformities impaired the patient’s ability to stand, so that the effect of treatment on gait and stability was not assessed. After 1 year of burosumab (10 mg every two weeks), increased serum phosphate levels up to 4.4 mg/dL and reduced ALP activity up to 1490 IU/L and BALP up to 608 μg/L was observed, while intact FGF23 levels remained elevated (243 pg/mL); serum levels of 25OHD and PTH were normal (31 ng/mL and 44 pg/mL, respectively). The dose of burosumab was maintained at 20 mg every two weeks despite increase of patient’s weight. Nonetheless, ALP activity and serum phosphate showed steady levels. X-ray examinations documented a slower but persistent progression of the FD, but the worsening of the femoral deformities required orthopedic surgery. Burosumab treatment was stopped before surgery (9 years and 9 months of chronological age). The post-surgical recovery period led to a prolongation of treatment stop up to 6 months, during which a pathological bilateral tibial fracture occurred. Before re-start of burosumab, ALP activity was increased and serum phosphate levels were reduced, but both rapidly improved during treatment (**Table 1**). During the period of treatment with burosumab no adverse events were observed.

**Table 1:** Overview of the biochemical parameters assessed before and after the treatment course with burosumab.

Laboratory tests *(reference range)*	Cholecalciferol and calcium carbonate *2 years 5 months*	Neridronate treatment *5 years 6 months*	Burosumab treatment *8 years 9 months*	Suspension of burosumab *9 years 9 months*	Restart of burosumab *10 years 5 months*	Last evaluation *11 years 7 months*
Total serum calcium *(9.3 - 10.6 mg/dL)*	10.4	10.1	10.0	10.2	10.0	9.8
Ionized calcium *(4.3 - 5.3 mg/dL)*	4.5	4.6	4.7	4.7	4.7	4.7
Phosphate *(*3.7-5.6 *mg/dL)*	4.4	3.6 ↓	3.1 ↓	4.4	3.2 ↓	4.0
ALP *(*105.0- 280.0*IU/L)*	660 ↑	1228 ↑	2118 ↑	1490 ↑	3385 ↑	1610 ↑
BALP *(*24.5-169.5 *μg/L)*	283 ↑	622 ↑	942 ↑	508 ↑	684 ↑	514 ↑
FGF23 *(23.2 - 95.4 pg/mL)*	–	144 ↑	251 ↑	243 ↑	463 ↑	222 ↑
25OHD *(20–100 ng/mL)*	29.0	37.0	58.0	31.0	22.0	26.0
PTH *(12–60 pg/mL)*	25.3	45.5	38.0	44.0	63.9 ↑	83.0 ↑
TmPO_4_/GFR *(3.4 - 5.8 mg/dL)*	–	3.3 ↓	2.8 ↓	3.3 ↓	2.9 ↓	3.7

ALP, alkaline phosphatase; BALP, bone isoenzyme of ALP; FGF23, fibroblast growth factor 23; 25OHD, 25-hydroxyvitamin D; PTH, parathyroid hormone; TmPO4/GFR, tubular maximum reabsorption of phosphate to glomerular filtration rate ratio."↓" means “under the reference range” and "↑" means “above the reference range”.

## Discussion

Data on the use of burosumab in patients with MAS and CSHS are few and limited to case reports. A phase 2 clinical trial concerning the use of burosumab in FD is still ongoing (NCT05509595).

Gladding et al. (26) reported a case of MAS with polyostotic FD and rachitic changes in a child treated with burosumab (0.8 mg/kg every two weeks) for 17 months with improvement of bone pain and stamina and reduction of occurrence of pathological fractures. Apperley et al. (27) described a 13 years old boy with FD, PPP, GH excess and hyperprolactinemia who underwent a 5 weeks course of burosumab (0.5 mg/kg every two weeks). The patient showed a normalization of bone markers after 5 weeks from the first dose of burosumab. Moreover, Sawamura et al. (28) described a 11 years old girl treated with burosumab (0.8 mg/kg every 2 weeks) for 1 year; after 3 months of treatment, the patient showed an improvement of clinical symptoms and reduction of radiolucency of the skeletal lesions. Data about the use of burosumab from previous cases reported in literature are summarized in **Table 2**.

**Table 2:** Posology and effects on clinical course and bone markers of burosumab treatment in patients with McCune-Albright syndrome from previously reported cases and in our patient.

Case	Dose	Duration	Serum phosphate levels	ALP activity	Pain control	Fractures and bone health
Gladding et al. (22)	20 mg every 2 weeks	17 months	Normalization	Improvement	Improvement	No fractures, no additional surgeries
Apperley et al. (23)	0.5 mg/kg every 2 weeks	5 weeks	Normalization	Normalization	Not reported	No fractures
Sawamura et al. (24)	0.8 mg/kg every 2 weeks	12 months	Normalization	Improvement	Improvement	No fractures, improvement of bone quality and reduced calcification within multiple fibrous lesions
Present case	0.8 mg/kg every 2 weeks	20 months	Lower limit of normalower limit of reference	Improvement	No pain	No fractures. Orthopedic surgery due progression of femoral deformities

ALP, alkaline phosphatase.

Similar observations were reported for the off-label use of burosumab to treat bone involvement in CSHS. Merz et al. (29), in a 3-year-old girl with CSHS who did not respond to conventional therapy, showed that a low dose of burosumab (0.3–0.4 mg/kg every 2 weeks) was associated with normalization of serum phosphate and ALP levels, improvement of rickets, partial recovery of Looser zones, as well as increased mobility and quality of life. Sugarman et al. (30) reported two patients with CSHS, a child and an adult, treated with burosumab (0.3 mg/kg every 3 weeks); both patients experienced improvement in clinical course of limb deformities, fractures, mobility, and laboratory parameters as phosphate levels and some markers of bone turnover. Khadora et al. (31) described a girl with CSHS treated with burosumab (0.3 mg/kg to 0.4 mg/kg) administered every two weeks for 12 months, who showed the normalization of the biochemical parameters, regression of rickets, partial healing of pseudofractures, improvement in the dysplastic skeletal lesions and perceived fatigue. Furthermore, Huyhn et al. (32) reported that burosumab treatment in a 15-year-old male with CSHS was associated with normalization of serum phosphate and ALP levels, and improvement of serum 1,25(OH)_2_D levels, muscular strength and pain. In a recent study of Abebe et al. (33) burosumab treatment (0.2 mg/kg every 2 weeks) over a period of 26 months in a 3-year-old patient with CSHS led to normalization of serum levels of phosphate, ALP and 1,25(OH)_2_D, with improvement in lower limb deformity, pain, growth delay, and bone lesions. Finally, da Silva et al. (34) described a case of CSHS with short stature, scoliosis and genu valgum who underwent treatment with burosumab at the age of 12 (0.8 mg/kg every 2 weeks) showing an improvement of mobility and pain associated with increased serum phosphate levels and a rapid decline in ALP levels after about 5 months since start of burosumab.

We described the clinical course of a patient with MAS and severe FD treated with burosumab for 20 months who showed some beneficial effects on bone metabolism. Some differences from the biochemical pattern reported in XLH patients treated with burosumab were evident (35–37). In children with XLH, serum phosphate levels usually increase up to low-normal values with a normalization of ALP activity (35–37). In our patient, ALP levels were reduced to approximately 50% of baseline values during the second course of treatment but never reached the normal range, as also reported in an adult with MAS and hypophosphatemia treated with burosumab (38). Likewise, FGF23 levels were persistently elevated during burosumab, despite the normalization of serum phosphate levels and the improvement of TmPO_4_/GFR ratio.

Our results are consistent with the origin of FD in MAS. In this condition, the constitutional activation of Gsα leading to the overproduction of FGF23 was not stopped by burosumab. Therefore, burosumab may improve phosphate metabolism, but the trigger to bone remodeling may persist. This would explain why bone deformities did not improve during burosumab, and its interruption led to a severe worsening of ALP. This finding suggests that the beneficial effect of burosumab on FD may be strictly related to phosphate metabolism and did not persist when the treatment was stopped. Some studies reported that FGF23-mediated hypophosphatemia was associated with a higher prevalence of FD-related complications, including fractures, scoliosis, and skull base deformities (12). In addition, the progression of FD lesions during treatment may indicate that the increased phosphate wasting could be a worsening factor of bone metabolism in MAS but not the key determinant of disease course.

In our patient, serum PTH levels were above the upper limit of normal after restarting burosumab treatment but normocalcemia was maintained.

In XLH patients undergoing conventional treatment with phosphate supplements and active vitamin D metabolites, secondary and tertiary hyperparathyroidism may occur (39, 40); however, data about potential occurrence of this complication in patients treated with burosumab are poor (41). Therefore, measurement of serum PTH levels in patients with MAS treated with burosumab should be recommended to identify the early occurrence of secondary or tertiary hyperparathyroidism.

In our patient the dose of burosumab was based on the approved dosing in Europe for patients with XLH (42). The dose of 20 mg every two weeks was not modified during the period of treatment as persistent reduction of ALP activity and normalization of serum phosphate levels were achieved. Similar results were found by Gladding et al. (26), whereas Apperley et al. (27) reported a normalization of ALP activity and serum phosphate levels with a dose as low as 0.5 mg/kg every two weeks. These differences may be explained by the extension and severity of FD in our patient and in those described by Gladding et al. (26).

A comparison of clinical and laboratory response of MAS and CSHS to the use of burosumab highlights how much the bone involvement may be different in these two conditions. In patients with MAS, biochemical markers improve but do not always normalize (26) while in patients with CSHS burosumab may lead to a normalization of bone markers, including ALP (29, 30, 33, 34). Moreover, in patients with CSHS an improvement of bone lesions has been documented (29–31, 33, 34). These findings suggest that the aberrant activation of RAS/MAPK pathway may cause a more consistent effect of FGF23 on bone lesions compared to a lesser effect on progression of FD due to GSα. In this perspective, burosumab may play a role as bone metabolism improving factor in patients with MAS but also improve the disease progression in patients with CSHS.

## Conclusions

Our study describes, for the second time in Europe, the effects of burosumab in a child with MAS. Burosumab treatment was associated with a normalization of serum phosphate levels and a reduction in ALP activity, and with a partial improvement of FD. Serum PTH levels did not change. No adverse events were found during burosumab treatment. Further studies in a larger number of patients are needed to assess if burosumab may have a therapeutic role for treatment of patients with MAS.

## Data Availability

The original contributions presented in the study are included in the article/supplementary material. Further inquiries can be directed to the corresponding author.
